# Cost Consequence Analysis of Belimumab versus Standard of Care for the Management of Systemic Lupus Erythematosus in Saudi Arabia: A Retrospective Cohort Study

**DOI:** 10.3390/ijerph20031917

**Published:** 2023-01-20

**Authors:** Aseel Alsuwayegh, Ibrahim A. Almaghlouth, Majed Ali Almasaoud, Abdullah Sulaiman Alzaid, Adel Abdulaziz Alsuhaibani, Lyan Hassan Almana, Sara Mohammed Alabdulkareem, Joud Abdullah Abudahesh, Yazed AlRuthia

**Affiliations:** 1 Corporate Department of Pharmacy Services, King Saud University Medical City, Riyadh 11451, Saudi Arabia; 2 Department of Medicine, College of Medicine, King Saud University, Riyadh 11461, Saudi Arabia; 3 College of Medicine, King Saud University, Riyadh 11461, Saudi Arabia; 4 Department of Clinical Pharmacy, College of Pharmacy, King Saud University, Riyadh 11451, Saudi Arabia; 5 Pharmacoeconomics Research Unit, Department of Clinical Pharmacy, College of Pharmacy, King Saud University, Riyadh 11451, Saudi Arabia

**Keywords:** systemic lupus erythematosus, belimumab, cost effectiveness analysis, Saudi Arabia, lupus nephritis

## Abstract

Background: Belimumab use for the management of systemic lupus erythematosus (SLE) has been limited, in part due to its high acquisition cost relative to the standard of care (SoC) and the uncertainties about its cost-effectiveness. Therefore, the aim of this study was to compare the cost and effectiveness of belimumab versus the SoC alone for the management of SLE using real-world data from the perspective of public healthcare payers in Saudi Arabia. Methods: Data were retrieved from a national prospective cohort of SLE, Saudi Arabia. Adult SLE patients (≥18 yrs.) treated with belimumab plus the SoC or the SoC alone for at least six months were recruited. The effectiveness was measured using the Systemic Lupus Erythematosus Disease Activity Index 2000 (SLEDAI-2K). Unit costs for health services and prescription drugs were retrieved from the Saudi ministry of health. Nonparametric bootstrapping with inverse probability weighting was conducted to generate the 95% confidence limits for the cost and effectiveness. Results: A total of 15 patients on belimumab plus the SoC and 41 patients on the SoC alone met the inclusion criteria and were included in the analysis. The majority of patients were females (91.07%) with a mean age of 38 years. The mean difference in cost and SLEDAI-2K score reduction between belimumab versus the SoC were USD 5303.16 [95% CI: USD 2735.61–USD 7802.52] and 3.378 [95% CI: 1.769–6.831], respectively. Belimumab demonstrated better effectiveness but higher cost in 96% of the bootstrap cost-effectiveness distributions. Conclusion: Future studies should use more robust research designs and a larger sample size to confirm the findings of this study.

## 1. Introduction

Systemic lupus erythematosus (SLE) is a chronic autoimmune systemic disease with complex biological pathophysiology that can result in multiple organ involvement and in numerous immunological laboratory abnormalities and clinical manifestations [1]. The inflammation caused by SLE can result in cutaneous manifestations, arthritis, or even serious renal impairment and cardiovascular diseases [2,3]. Furthermore, SLE seems to follow one of three patterns: chronic active, relapsing–remitting, and, to a lesser extent, long quiescent course [4]. Although the survival rates for SLE patients have significantly improved over the past five decades, their risk of mortality is believed to be three to five times higher than the general healthy population [5]. Additionally, SLE patients suffer from reduced health-related quality of life (HRQoL) due to the substantial comorbidity that accompanies the disease progression [6,7]. Such an impact of the disease on various aspects of a patient’s life creates a sense of urgency to develop more effective treatments that are capable of controlling the disease and, consequently, improving the patients’ quality of life, productivity, and long-term outcomes [8,9]. 

In recent decades, SLE treatment has moved from the use of hydroxychloroquine, systemic corticosteroids, and conventional immunosuppressive drugs to more targeted therapeutic agents [10]. Novel therapies targeting B-cell stimulating factors and interferon receptors have just been recently approved as treatment options for patients with SLE, with more molecules in the pipelines [9,10]. Belimumab is a B-cell stimulating factor inhibitor that emerged as an effective add-on treatment option for SLE patients with active SLE [11,12]. The initial FDA approval was granted for those with the active disease (mainly mucocutaneus and musculoskeletal involvement), despite the use of the standard of care treatment. However, this was later extended to those with lupus nephritis based on belimumab efficacy that was demonstrated in BLISS-LN [13,14]. Unfortunately, the adoption of such effective treatment to address the unmet need in clinical practice faces some challenges that might be related to the high acquisition cost relative to the standard of care (SoC) and the uncertainties about its cost-effectiveness, especially in emerging markets, such as Saudi Arabia. No study has compared the cost-effectiveness of belimumab to the standard of care for the management of SLE in Saudi Arabia using real-world data, despite the multiple studies that examined its cost-effectiveness in different healthcare settings [15,16,17]. Therefore, the aim of this study was to compare the cost and effectiveness of belimumab versus the standard of care for the management of SLE in Saudi Arabia using real-world data.

## 2. Methods

### 2.1. Study Design and Population

This was a retrospective cohort study that was conducted at King Khalid University hospital, which is a university-affiliated tertiary care center in Riyadh, Saudi Arabia, using a national prospective cohort of SLE [18]. The study took place between 21 March 2020 and 30 December 2021. Adult patients (i.e., ≥18 yrs.) with SLE managed by the standard of care (i.e., corticosteroids and hydroxychloroquine) or belimumab plus the standard of care, and who were treated for at least six months were included in the analysis. Pediatric patients under 18 years of age, those with cancer, both hematologic and solid malignancies, chronic kidney disease, cardiovascular disease, and those who have suffered a stroke were excluded. The included data elements were: age, gender, duration of illness in years, comorbid medical conditions (e.g., diabetes, dyslipidemia, hypertension, etc.), prescription medications, hospital admissions and length of stay, lab tests, imaging studies, outpatient visits, and disease activity as assessed by the Systemic Lupus Erythematosus Disease Activity Index 2000 (SLEDAI-2K) [18,19]. The SLEDAI-2K is a modified version of the SLEDAI that was developed in 1985 as a clinical measure for the assessment of lupus disease activity in the preceding 10 days [20]. It consists of 24 clinical and laboratory variables that are weighted by the type of manifestation, and is completed by physicians and needs clinical examination and laboratory test results to be scored [19]. SLE disease activity has been categorized based on SLEDAI-2K scores as follows: no activity (SLEDAI-2K = 0), mild activity (SLEDAI-2K = 1–5), moderate activity (SLEDAI-2K = 6–10), high activity (SLEDAI-2K = 11–19), and very-high activity (SLEDAI ≥ 20) [21].

### 2.2. Statistical Analysis 

The baseline characteristics of the patients were presented using mean, standard deviation, frequencies, and percentages. Student’s t-test, a one-way ANOVA, a chi-square test, and a Fisher’s exact test were conducted, as appropriate, to compare the baseline characteristics of the patients on belimumab-based treatment versus their counterparts on the standard of care (SoC). The outcome was defined as a reduction in the SLEDAI-2K score, while costs included all outpatient and inpatient services (e.g., prescription drugs, lab tests, imaging studies, hospitalization, clinic visits, emergency department visits, etc.). A paired t-test was conducted to compare the difference in SLEDAI-2k scores pre- and post-treatment with belimumab and the SoC (corticosteroids and hydroxychloroquine). In order to generate the 95% confidence limits for the mean difference in outcome and cost, non-parametric bootstrapping with 10,000 replications was conducted. The bootstrap was used to simulate what the results might be if the comparison between belimumab-based treatment and SoC was repeated over and over again, and it is a powerful tool to avoid parametric assumptions when computing confidence intervals to examine the uncertainty about the outcomes, which in this case are the difference in costs and the SLEDAI-2K score. The SAS macro enables such time-consuming and robust methods to be conducted [22]. To adjust for selection bias, inverse probability treatment weighting was used, which controlled for age, gender, baseline SLEDAI-2K score, duration of illness, and duration of therapy. Moreover, multiple linear regression was conducted to examine the impact of belimumab versus the SoC on the SLEDAI-2K score difference (baseline SLEDAI-2K score–follow-up SLEDAI-2K score) controlling for age, gender, baseline SLEDAI-2K score, treatment duration, and duration of illness. The minimum sample size was estimated to be 52 patients based on α = 0.05, β = 0.8, power of 0.8, effect size of (d) = 0.85, and an allocation ratio of 3 to 1 for those on the SoC versus their counterparts on the belimumab-based treatment.

### 2.3. Ethical Considerations 

The study did not involve any collection of biological samples or medical interventions and only included an EMR review. Therefore, the institutional review board has waived the requirement for a written informed consent form. The study was approved by the institutional review board of King Saud University College of Medicine on 21 March 2021 (IRB Approval of Research Project No. E-21-5775). No personal identifiers (e.g., name, medical record number, national identification number, telephone, etc.) were collected and the study adhered to the ethical principles of the Helsinki declaration [23].

## 3. Results

Although more than 400 medical records for patients with different autoimmune disorders have been reviewed, only 56 patients have met the inclusion criteria and were included in the analysis. Of those, 15 patients were on belimumab-based treatment and 41 patients were on the SoC (e.g., hydroxychloroquine and/or prednisone). Although SLE patients on belimumab were four years younger than their counterparts on the SoC, this difference was not statistically significant (34.54 vs. 39.93 yrs., *p*-value = 0.0861). The majority of patients were females (91.07%) and the mean duration of illness was 13 years. Patients’ mean duration of treatment was approximately 10 months with no difference between the patients on belimumab-based treatment and their counterparts on the SoC. SLE manifestations, such as proteinuria, hematuria, leukopenia, thrombocytopenia, and myositis, were not present among more than 15% of the patients, with no significant difference between the belimumab-based treatment and the SoC. However, arthritis and lupus nephritis were present among more than 40% of the patients, with arthritis being present among more than 60% of patients on belimumab-based treatment, while lupus nephritis was present among 51% of the patients on the SoC. Although alopecia was only present among 16% of the patients, about one-third of the patients on belimumab had alopecia. Most of the patients on belimumab had chronic cutaneous lupus (i.e., 60%) and acute or subacute cutaneous lupus (i.e., 53%). On the other hand, most of the patients on the standard of care had no or mild disease activity (i.e., 61%) at baseline according to the SLEDAI-2K score. About two-thirds of the patients on belimumab (i.e., 67%) had moderate or high disease activity at baseline according to the SLEDAI-2K score as shown in Table 1.

**Figure 1:**
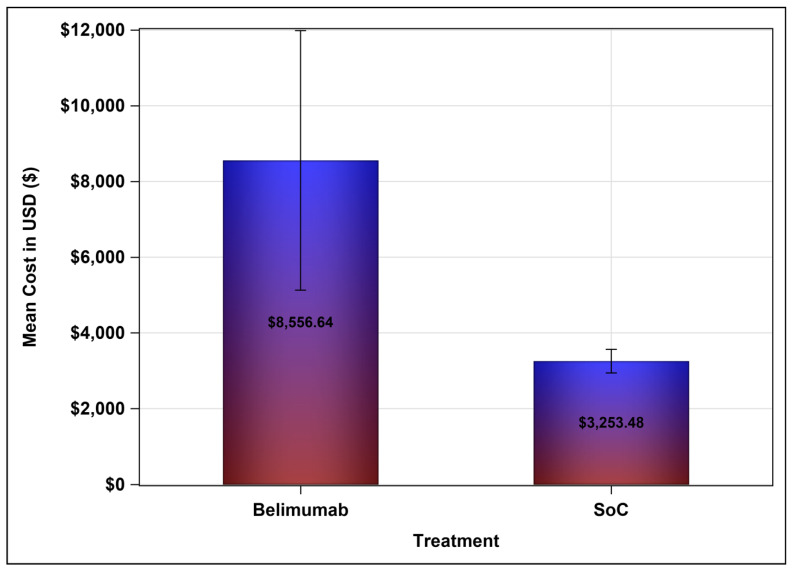
Mean treatment costs of belimumab-based treatment regimens and the standard of care (SoC).

**Figure 2:**
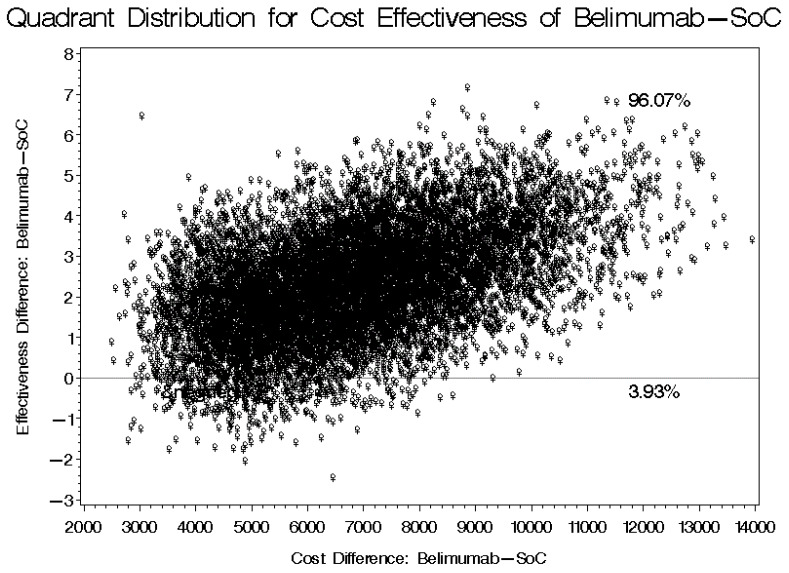
Bootstrap distribution of cost-effectiveness for the belimumab-based treatment regimen versus the standard of care (SoC) for patients at the annual screening.

**Figure 3:**
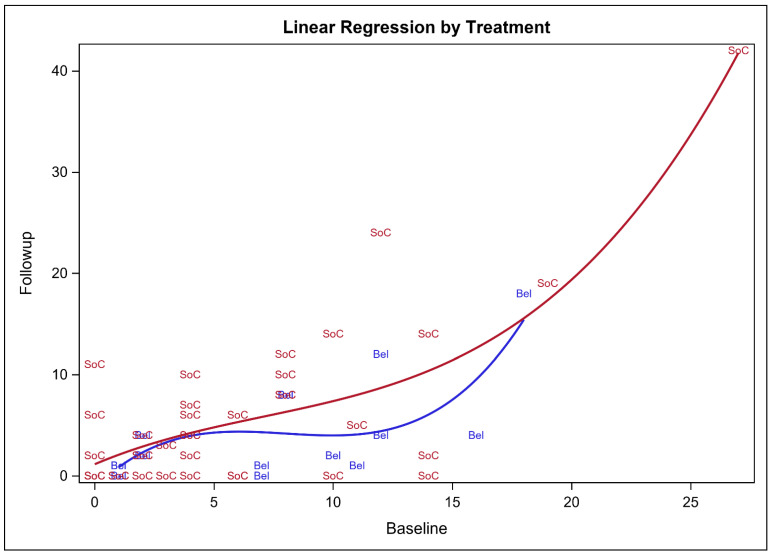
The regression line for the relationship between baseline and follow-up SLEDAI-2K scores for the patients on belimumab (Bel) and the standard of care (SoC).

The mean direct medical cost (e.g., lab tests, clinic visits, imaging studies, prescription drugs, etc.) for patients on belimumab-based treatment was USD 8556.64, in comparison to USD 3253.48 for patients on the SoC, as shown in Figure 1. The mean difference in cost between the belimumab-based treatment and the SoC was USD 5303.16 with a 95% bootstrap confidence interval of USD 2735.61–USD 7802.52, which means that the use of belimumab will result in a higher overall direct medical cost, mainly due to its higher acquisition cost. On the other hand, the SLEDAI-2K score, which measures the disease activity, for patients on belimumab was reduced on average by 3.33 points after at least six months of treatment (*p* = 0.0139). On the contrary, patients on the SoC (e.g., corticosteroids and hydroxychloroquine) did not have a reduction in their SLEDAI-2K scores and had their scores increased on average by 0.0487 after at least six months of follow-up, however, this was not statistically significant (*p* = 0.954). These findings suggest a progression or no change in the disease activity after at least six months of follow-up for patients who were treated with the SoC. The 95% bootstrap confidence interval for the mean difference in the SLEDAI-2K scores between patients on belimumab-based treatment and the SoC ranged from 1.769 to 6.831, as shown in Table 2. According to the bootstrap cost-effectiveness distributions that are shown in Figure 2, the use of belimumab will result in a greater reduction in the SLEDAI-2K score after at least six months of treatment, in comparison to the SoC in more than 96% of the bootstrap cost effectiveness distributions, but with a higher cost. On the other hand, the use of belimumab might result in a higher cost and worse clinical outcomes (e.g., higher SLEDAI-2K score) in almost 4% of the bootstrap cost-effectiveness distributions. This means that most of the dispersions or distributions of the bootstrap distributions (96%) are in the right upper quadrant of the cost-effectiveness plane (more effective but costlier) and only 4% of the distributions are in the left upper quadrant (less effective and costlier). Patients treated with belimumab with relatively high baseline SLEDAI-2K scores (≥10) tend to have lower SLEDAI-2K scores at the follow-up (<10); however, this trend was not observed among patients on the SoC, as demonstrated in Figure 3. Moreover, the use of belimumab was associated with a significant reduction in the SLEDAI-2K score at the follow-up (β-estimate = 3.640, 95% confidence interval [0.1573–7.122], *p* = 0.0409), controlling for age, gender, baseline SLEDAI-2K score, duration of therapy and duration of illness (Table 3).

## 4. Discussion

The utilization rate of biological therapies, such as belimumab, for the management of SLE has been increasing [24,25]. In this study, belimumab plus the SoC was found to be more effective than the SoC alone in reducing the SLEDAI-2K score. These findings confirm the findings of most clinical trials that demonstrated a positive impact of belimumab on SLE disease activity and HRQoL [26]. However, this comes with a significantly higher cost than the SoC alone. The mean difference in the treatment cost between belimumab-based therapy and the SoC was USD 5303.16, with a 95% confidence limit ranging from USD 2735.61 to USD 7802.52, while the mean difference in the SLEDAI-2K score reduction between the two treatment groups was 3.38 with a 95% confidence limit ranging from 1.76 to 6.83. This can be translated into USD 1569.91 per one-point reduction in the SLEDAI-2K. While an initial upfront cost can be seen with the use of belimumab, a considerable cost avoidance also needed to be addressed. In this study, we demonstrated that belimumab was associated with a 3.38 reduction in the SLEDAI-2K score. The reduction in the SLEDAI score has been associated with a significant reduction in damage accrual [27]. This was beautifully demonstrated in the Bruce IN et al. study, in which, for a three-point increase in SLEDAI, there was about a 25% increase in the rate of damage progression from damage-naïve patients to a more accrual damage state [27]. The utilization of belimumab has also shown to be associated with lower damage accrual in a large propensity score-matched cohort study by Urowitz et al. [28]. This raises an important question of whether the accumulation of damage in SLE can lead to an increase in disease management costs. While data on healthcare utilization costs in SLE are not abundant, Samnaliev et al. demonstrated an overall increase in all-cause healthcare costs following an SLE diagnosis. Such an increase continued in those with moderate-to-severe disease, with up to a four-fold increase in those with severe disease who were followed up for 10 years following diagnosis [29]. This increase in direct medical cost might be related to the higher utilization rates of inpatient services, medications, or perhaps accrual damage [29]. 

Belimumab was found to be cost effective in several economic evaluations [15,16,17]. According to a study that examined the cost-effectiveness of belimumab and voclosporin for the management of SLE among patients with lupus nephritis in the United States (U.S.), which included four health states and data from pivotal clinical trials, belimumab but not voclosporin was found to be cost effective in comparison to the SoC at a willingness-to-pay threshold of USD 100,000 per quality-adjusted life year (QALY) [17]. However, this economic evaluation was largely based on short-term data from clinical trials, which do not necessarily represent patients in real-world settings. In addition, the study used the assumption of sustained clinical effect for three years, despite the shorter follow-up periods in the utilized data for clinical trials [17]. Similarly, a microsimulation model from the United Kingdom (UK) that was adapted to the Spanish settings and examined the cost-effectiveness of belimumab plus the SoC versus the SoC alone for the management of SLE from the societal perspective found belimumab to be cost effective using the cost-effectiveness threshold of EUR 30,000/QALY. However, the study extrapolated data from clinical trials and used utility values in their lifetime horizon analysis from the UK and the U.S., as well as some direct and indirect cost data from Spain [16]. These limitations in the published cost-effectiveness analyses highlight the uncertainties about the cost effectiveness of belimumab for the management of SLE. This highlights the importance of cost effectiveness analyses that are based on real-world data, which is the case in this study. Additionally, our findings were further substantiated by a multiple regression analysis that controlled for potential confounders, such as baseline SLEDAI-2K scores, age, gender, duration of treatment, and duration of illness [30,31].

Although this study used real-world local data in contrast to other published cost-effectiveness analyses that compared belimumab to the SoC for SLE management using hypothetical cohorts, it has multiple limitations that must be acknowledged. First, the sample size was very small and the data were retrieved from a single center, which limits the generalizability of the findings. This low rate of biologics utilization among SLE patients is consistent with a previously published study that examined the drug utilization patterns and economic burden of SLE in the U.S. based on the claims data of two large administrative databases for privately insured individuals [32]. We believe that our work is the first step toward a full economic evaluation to assess the costs and consequences of different biologic therapies for SLE in our region with a clear plan to expand this work in the future using larger datasets from multiple centers. 

Secondly, this study did not examine the cost-effectiveness of belimumab with regard to the improvement of HRQoL as most economic analyses do, [15,16,17] and used the SLEDAI-2K score reduction as the study’s effectiveness outcome. This was largely related to the lack of formal assessment of HRQoL before and after the treatment in our cohort. However, SLEDAI-2K is commonly used to assess the disease activity in clinical practice and has been used in assessing the effectiveness of belimumab in multiple observational studies [24,33]. Moreover, validated health state utility estimates for the Saudi population do not exist so far, and the use of utility estimates from other countries does not reflect the actual utility values for the studied patient population [34]. Additionally, the study did not include the Cutaneous Lupus Erythematosus Disease Area and Severity Index (CLASI) or the Systemic Lupus International Collaborating Clinics (SLICC) to examine the severity of the disease and assess the patients’ responsiveness to therapy [35,36]. Thirdly, no sensitivity analyses were conducted with regard to varying the acquisition cost of prescription drugs including belimumab. This has not been conducted since there is not a validated cost-effectiveness threshold for Saudi Arabia; therefore, the authors opted to present the actual cost and consequences of both belimumab-based treatment and the SoC for SLE from the perspective of public healthcare payers in Saudi Arabia, which represents nearly 60% of the overall Saudi healthcare market [34]. Finally, the study did not capture the indirect costs and only included the direct medical costs since it was conducted from the perspective of public healthcare payers; however, further work is planned to expand on this particular area since it may provide a more comprehensive view of the true economic burden and utility of belimumab in the lupus population. 

## 5. Conclusions

Belimumab is an effective biologic therapy for the management of SLE and reducing accrual damage [37,38]. Therefore, examining the cost-effectiveness of SLE treatments in delaying disease progression and improving clinical outcomes among patients from different ethnic groups is important. In this study, belimumab has demonstrated better effectiveness in combination with the SoC in comparison to the SoC alone with a higher upfront direct cost and potentially long-term cost avoidance. Larger studies are needed to confirm these findings.

## Figures and Tables

**Table 1:** Patients’ baseline characteristics.

Characteristic	Belimumab (*n* = 15)	Standard of Care (SoC) (*n* = 41)	*p*-Value	Total (*n* = 56)
Age in yrs., (mean ± SD)	34.54 ± 7.70	39.93 ± 10.95	0.0861	38.48 ± 10.40
Gender, (*n*, %)				
Male	1 (6.67)	4 (9.76)	0.7196	5 (8.93)
Female	14 (93.33)	37 (90.24)		51 (91.07)
Disease duration in years, (mean ± SD)	12.40 ± 7.05	13.37 ± 6.02	0.6118	13.11 ± 6.26
Duration of therapy in months, (mean ± SD)	10.00 ± 7.19	9.97 ± 3.03	0.9900	9.98 ± 4.45
Proteinuria, (*n*, %)	1 (6.67)	5 (12.20)	0.5536	6 (10.71)
Hypertension (HTN), (*n*, %)	1 (6.67)	6 (14.63)	0.5879	7 (12.50)
Hematuria, (*n*, %)	1 (6.67)	2 (4.88)	0.7924	3 (5.36)
Hemolytic anemia, (*n*, %)	2 (13.33)	1 (2.44)	0.201	3 (5.36)
Leukopenia, (*n*, %)	1 (6.67)	7 (17.07)	0.4276	8 (14.29)
Arthritis, (*n*, %)	10 (66.67)	17 (41.46)	0.1334	27 (48.21)
Serositis, (*n*, %)	1 (6.67)	3 (7.32)	0.9333	4 (7.14)
Lupus nephritis, (*n*, %)	4 (26.67)	21 (51.22)	0.0902	25 (44.64)
Thrombocytopenia, (*n*, %)	1 (6.67)	6 (14.63)	0.6607	7 (12.50)
Diabetes, (*n*, %)	0 (0.00)	3 (7.32)	0.5563	3 (5.36)
Dyslipidemia, (*n*, %)	2 (13.33)	4 (9.76)	0.7793	6 (10.71)
Alopecia, (*n*, %)	5 (33.33)	4 (9.76)	0.0479	9 (16.07)
Myositis, (*n*, %)	1 (6.67)	3 (7.32)	0.9333	4 (7.14)
Chronic cutaneous lupus, (*n*, %)	9 (60.00)	5 (12.20)	0.0011	14 (25.00)
Acute or subacute cutaneous lupus, (*n*, %)	8 (53.33)	12 (29.27)	0.1218	20 (35.71)
Baseline systemic lupus erythematosus disease activity index (SLEDAI-2K)				
No activity (SLEDAI = 0)	0 (0.00)	10 (24.39)	0.1598	10 (17.86)
Mild activity (SLEDAI = 1 to 5)	5 (33.33)	15 (36.59)		20 (35.71)
Moderate activity (SLEDAI = 6 to 10)	5 (33.33)	9 (21.95)		14 (25.00)
High activity (SLEDAI = 11 to 19)	5 (33.33)	6 (14.63)		11 (19.64)
Very high activity (SLEDAI ≥ 20)	0 (0.00)	1 (2.44)		1 (1.79)

**Table 2:** The mean Systemic Lupus Erythematosus Disease Activity Index 2000 (SLEDAI–2K) score difference between the baseline and follow-up period and treatment costs for a belimumab-based treatment regimen versus the standard of care (SoC).

	Belimumab	SoC	Mean Difference (95% Confidence Interval)
Cost of treatment (USD), mean ± SD	8556.64 ± 6189.75	3253.48 ± 987.86	5303.16 (2735.61–7802.52)
Difference between baseline and follow–up SLEDAI–2K score, mean ± SD	3.33 ± 4.59	−0.0487 ± 5.48	3.378 (1.769–6.831)

**Table 3:** Multiple regression analysis for the relationship between the SLEDAI-2K score difference (baseline SLEDAI-2K score–follow-up SLEDAI-2K score) and the use of belimumab versus the standard of care (SoC) for SLE treatment.

Variable	Regression Coefficient (β-Estimate)	*p*-Value	95% Confidence Interval
Belimumab vs. SoC	3.6400	0.0409 *	0.15735–7.1227
Baseline SLEDAI-2K score	0.0932	0.4647	−0.1610–0.3474
Duration of treatment	0.9336	0.3079	−0.8873–2.7546
Age	0.0418	0.5736	−0.1065–0.1902
Female vs. male	1.5707	0.5496	−3.6681–6.8095
Duration of illness	−0.6195	0.5796	−2.8520–1.6130

* *p*-value < 0.05.

## Data Availability

The data are available upon reasonable request from the corresponding authors (Yazed AlRuthia and Ibrahim A. Almaghlouth).
